# The use of evidence to guide decision-making during the COVID-19 pandemic: divergent perspectives from a qualitative case study in British Columbia, Canada

**DOI:** 10.1186/s12961-024-01146-2

**Published:** 2024-06-03

**Authors:** Laura Jane Brubacher, Chris Y. Lovato, Veena Sriram, Michael Cheng, Peter Berman

**Affiliations:** 1 School of Population and Public Health, University of British Columbia, Vancouver, Canada; 2 School of Public Health Sciences, University of Waterloo, Waterloo, Canada; 3 School of Public Policy and Global Affairs, University of British Columbia, Vancouver, Canada

**Keywords:** Decision-making, Evidence, COVID-19, Public health, Policy-making, Canada, Qualitative

## Abstract

**Background:**

The challenges of evidence-informed decision-making in a public health emergency have never been so notable as during the COVID-19 pandemic. Questions about the decision-making process, including what forms of evidence were used, and how evidence informed—or did not inform—policy have been debated.

**Methods:**

We examined decision-makers' observations on evidence-use in early COVID-19 policy-making in British Columbia (BC), Canada through a qualitative case study. From July 2021- January 2022, we conducted 18 semi-structured key informant interviews with BC elected officials, provincial and regional-level health officials, and civil society actors involved in the public health response. The questions focused on: (1) the use of evidence in policy-making; (2) the interface between researchers and policy-makers; and (3) key challenges perceived by respondents as barriers to applying evidence to COVID-19 policy decisions. Data were analyzed thematically, using a constant comparative method. Framework analysis was also employed to generate analytic insights across stakeholder perspectives.

**Results:**

Overall, while many actors’ impressions were that BC's early COVID-19 policy response was evidence-informed, an overarching theme was a lack of clarity and uncertainty as to *what* evidence was used and *how* it flowed into decision-making processes. Perspectives diverged on the relationship between 'government' and public health expertise, and whether or not public health actors had an independent voice in articulating evidence to inform pandemic governance. Respondents perceived a lack of coordination and continuity across data sources, and a lack of explicit guidelines on evidence-use in the decision-making process, which resulted in a sense of fragmentation. The tension between the processes involved in research and the need for rapid decision-making was perceived as a barrier to using evidence to inform policy.

**Conclusions:**

Areas to be considered in planning for future emergencies include: information flow between policy-makers and researchers, coordination of data collection and use, and transparency as to how decisions are made—all of which reflect a need to improve communication. Based on our findings, clear mechanisms and processes for channeling varied forms of evidence into decision-making need to be identified, and doing so will strengthen preparedness for future public health crises.

**Supplementary Information:**

The online version contains supplementary material available at 10.1186/s12961-024-01146-2.

## Background

The challenges of evidence-informed decision-making^1^ in a public health emergency have never been so salient as during the COVID-19 pandemic, given its unprecedented scale, rapidly evolving virology, and multitude of global information systems to gather, synthesize, and disseminate evidence on the SARS-CoV-2 virus and associated public health and social measures [1–3]. Early in the COVID-19 pandemic, rapid decision-making became central for governments globally as they grappled with crucial decisions for which there was limited evidence. Critical questions exist, in looking retrospectively at these decision-making processes and with an eye to strengthening future preparedness: Were decisions informed by 'evidence'? What forms of evidence were used, and how, by decision-makers? [4–6].

Scientific evidence, including primary research, epidemiologic research, and knowledge synthesis, is one among multiple competing influences that inform decision-making processes in an outbreak such as COVID-19 [7]. Indeed, the use of multiple forms of evidence has been particularly notable as it applies to COVID-19 policy-making. Emerging research has also documented the important influence of ‘non-scientific’ evidence such as specialized expertise and experience, contextual information, and level of available resources [8–10]. The COVID-19 pandemic has underscored the politics of evidence-use in policy-making [11]; what evidence is used and how can be unclear, and shaped by political bias [4, 5]. Moreover, while many governments have established scientific advisory boards, the perspectives of these advisors were reportedly largely absent from COVID-19 policy processes [6]. How evidence and public health policy interface—and intersect—is a complex question, particularly in the dynamic context of a public health emergency. 

Within Canada, a hallmark of the public health system and endorsed by government is evidence-informed decision-making [12]. In British Columbia (BC), Canada, during the early phases of COVID-19 (March—June 2020), provincial public health communication focused primarily on voluntary compliance with recommended public health and social measures, and on supporting those most affected by the pandemic. Later, the response shifted from voluntary compliance to mandatory enforceable government orders [13]. Like many other jurisdictions, the government’s public messaging in BC asserted that the province took an approach to managing the COVID-19 pandemic and developing related policy that was based on scientific evidence, specifically. For example, in March 2021, in announcing changes to vaccination plans, Dr. Bonnie Henry, the Provincial Health Officer, stated, "*This is science in action*" [14]. As a public health expert with scientific voice, the Provincial Health Officer has been empowered to speak on behalf of the BC government across the COVID-19 pandemic progression. While this suggests BC is a jurisdiction which has institutionalized scientifically-informed decision-making as a core tenet of effective public health crisis response, it remains unclear as to whether BC’s COVID-19 response could, in fact, be considered evidence-informed—particularly from the perspectives of those involved in pandemic decision-making and action. Moreover, if evidence-informed, what types of evidence were utilized and through what mechanisms, how did this evidence shape decision-making, and what challenges existed in moving evidence to policy and praxis in BC’s COVID-19 response?

The objectives of this study were: (1) to explore and characterize the perspectives of BC actors involved in the COVID-19 response with respect to evidence-use in COVID-19 decision-making; and (2) to identify opportunities for and barriers to evidence-informed decision-making in BC’s COVID-19 response, and more broadly. This inquiry may contribute to identifying opportunities for further strengthening the synthesis and application of evidence (considered broadly) to public health policy and decision-making, particularly in the context of future public health emergencies, both in British Columbia and other jurisdictions.

## Methods

### Study context

This qualitative study was conducted in the province of British Columbia (BC), Canada, a jurisdiction with a population of approximately five million people [15]. Within BC’s health sector, key actors involved in the policy response to COVID-19 included: elected officials, the BC Government’s Ministry of Health (MOH), the Provincial Health Services Authority (PHSA),^2^ the Office of the Provincial Health Officer (PHO),^3^ the BC Centre for Disease Control (BCCDC),^4^ and Medical Health Officers (MHOs) and Chief MHOs at regional and local levels.

Health research infrastructure within the province includes Michael Smith Health Research BC [16] and multiple post-secondary research and education institutions (e.g., The University of British Columbia). Unlike other provincial (e.g., Ontario) and international (e.g., UK) jurisdictions, BC did not establish an independent, formal scientific advisory panel or separate organizational structure for public health intelligence in COVID-19. That said, a Strategic Research Advisory Council was established, reporting to the MOH and PHO, to identify COVID-19 research gaps and commission needed research for use within the COVID-19 response [17].

This research was part of a multidisciplinary UBC case study investigating the upstream determinants of the COVID-19 response in British Columbia, particularly related to institutions, politics, and organizations and how these interfaced with, and affected, pandemic governance [18]. Ethics approval for this study was provided by the University of British Columbia (UBC)’s Institutional Research Ethics Board (Certificate #: H20-02136).

### Data collection

From July 2021 to January 2022, 18 semi-structured key informant interviews were conducted with BC elected officials, provincial and regional-level health officials, and civil society actors (e.g., within non-profit research organizations, unions) (Table 1). Initially, respondents were purposively sampled, based on their involvement in the COVID-19 response and their positioning within the health system organizational structure. Snowball sampling was used to identify additional respondents, with the intent of representing a range of organizational roles and actor perspectives. Participants were recruited via email invitation and provided written informed consent to participate.

**Table 1:** Number of actors who were interviewed, by organizational role or position in the pandemic response

Role/Position	Participant ID
1. Provincial-level health officials (n = 11)	IDI1, IDI2, IDI3, IDI4, IDI5, IDI7, IDI8*, IDI9, IDI12, IDI13*, IDI16
2. Regional-level health officials (n = 2)	IDI6, IDI10
3. Elected officials (n = 1)	IDI15
4. Civil society actors (n = 4)	IDI11, IDI14, IDI17, IDI18

*Former role (retired)

Interviews were conducted virtually using Zoom® videoconferencing, with the exception of one hybrid in-person/Zoom® interview. Each interview was approximately one hour in duration. One to two research team members led each interview. The full interview protocol focused on actors’ descriptions of decision-making processes across the COVID-19 pandemic progression, from January 2020 to the date of the interviews, and they were asked to identify key decision points (e.g., emergency declaration, business closures) [see Additional File 1 for the full semi-structured interview guide]. For this study, we used a subset of interview questions focused on evidence-use in the decision-making process, and the organizational structures or actors involved, in BC's early COVID-19 pandemic response (March–August 2020). Questions were adapted to be relevant to a respondent’s expertise and particular involvement in the response. ‘Evidence’ was left undefined and considered broadly by the research team (i.e., both ‘scientific’/research-based and ‘non-scientific’ inputs) within interview questions, and therefore at the discretion of the participant as to what inputs they perceived and described as ‘evidence’ that informed or did not inform pandemic decision-making. Interviews were audio-recorded over Zoom® with permission and transcribed using *NVivo Release 1.5©* software. Each transcript was then manually verified for accuracy by 1–2 members of the research team.

### Data analysis

An inductive thematic analysis was conducted, using a constant comparative method, to explore points of divergence and convergence across interviews and stakeholder perspectives [19]. Transcripts were inductively coded in *NVivo Release 1.5©* software, which was used to further organize and consolidate codes, generate a parsimonious codebook to fit the data, and retrieve interview excerpts [20]. Framework analysis was also employed as an additional method for generating analytic insights across stakeholder perspectives and contributed to refining the overall coding [21]. Triangulation across respondents and analytic methods, as well as team collaboration in reviewing and refining the codebook, contributed to validity of the analysis [22].

## Results

### How did evidence inform early COVID-19 policy-making in BC?

Decision-makers described their perceptions on the use of evidence in policy-making; the interface between researchers and policy-makers; and specific barriers to evidence-use in policy-making within BC’s COVID-19 response. In discussing the use of evidence, respondents focused on ‘scientific’ evidence; however, they noted a lack of clarity as to *how* and *what* evidence flowed into decision-making. They also acknowledged that ‘scientific’ evidence was one of multiple factors influencing decisions. The themes described below reflect the narrative underlying their perspectives.

### Perceptions of evidence-use

Multiple provincial actors generally expressed confidence or had an overall impression that decisions were evidence-based (IDI5,9), stating definitively that, *"I don’t think there was a decision we made that wasn’t evidence-informed"* (IDI9) and that *"the science became a driver of decisions that were made"* (IDI5). However, at the regional health authority level, one actor voiced skepticism that policy decisions were consistently informed by scientific evidence specifically, stating, *"a lot of decisions [the PHO] made were in contrast to science and then shifted to be by the science" (*IDI6). The evolving nature of the available evidence and scientific understanding of the virus throughout the pandemic was acknowledged. For instance, one actor stated that, *"I’ll say the response has been driven by the science; the science has been changing…from what I’ve seen, [it] has been a very science-based response"* (IDI3).

Some actors narrowed in on certain policy decisions they believed were or were not evidence-informed. Policy decisions in 2020 that actors believed were directly informed by scientific data included the early decision to restrict informal, household gatherings; to keep schools open for in-person learning; to implement a business safety plan requirement across the province; and to delay the second vaccine dose for maximum efficacy. One provincial public health actor noted that an early 2020 decision made, within local jurisdictions, to close playgrounds was not based on scientific evidence. Further, the decision prompted public health decision-makers to centralize some decision-making to the provincial level, to address decisions being made 'on the ground' that were not based on scientific evidence (IDI16). Similarly, they added that the policy decision to require masking in schools was not based on scientific evidence; rather, *"it's policy informed by the noise of your community."* As parents and other groups within the community pushed for masking, this was *"a policy decision to help schools stay open."*

Early in the pandemic response, case data in local jurisdictions were reportedly used for monitoring and planning. These *"numerator data"* (IDI1), for instance case or hospitalization counts, were identified as being the primary mode of evidence used to inform decisions related to the implementation or easing of public health and social measures. The ability to generate epidemiological count data early in the pandemic due to efficient scaling up of PCR testing for COVID-19 was noted as a key advantage (IDI16). As the pandemic evolved in 2020, however, perspectives diverged in relation to the type of data that decision-makers relied on. For example, it was noted that BCCDC administered an online, voluntary survey to monitor unintended consequences of public health and social measures and inform targeted interventions. Opinions varied on whether this evidence was successfully *applied* in decision-making. One respondent emphasized this lack of application of evidence and perceived that public health orders were not informed by the level and type of evidence available, beyond case counts: *"[In] a communicable disease crisis like a pandemic, the collateral impact slash damage is important and if you're going to be a public health institute, you actually have to bring those to the front, not just count cases"* (IDI1).

There also existed some uncertainty and a perceived lack of transparency or clarity as to how or whether data analytic ‘entities’, such as BCCDC or research institutions, fed directly into decision-making. As a research actor shared, *"I’m not sure that I know quite what all those channels really look like…I’m sure that there’s a lot of improvement that could be driven in terms of how we bring strong evidence to actual policy and practice"* (IDI14). Another actor explicitly named the way information flowed into decision-making in the province as *"organic"* (IDI7). They also noted the lack of a formal, independent science advisory panel for BC’s COVID-19 response, which existed in other provincial and international jurisdictions. Relatedly, one regional health authority actor perceived that the committee that was convened to advise the province on research, and established for the purpose of applying research to the COVID-19 response, *"should have focused more on knowledge translation, but too much time was spent commissioning research and asking what kinds of questions we needed to ask rather than looking at what was happening in other jurisdictions"* (IDI6). Overall, multiple actors noted a lack of clarity around application of evidence and who is responsible for ensuring evidence is applied. As a BCCDC actor expressed, in relation to how to prevent transmission of COVID-19:

We probably knew most of the things that we needed to know about May of last year [2020]. So, to me, it’s not even what evidence you need to know about, but who’s responsible for making sure that you actually apply the evidence to the intervention? Because so many of our interventions have been driven by peer pressure and public expectation rather than what we know to be the case [scientifically] (IDI1).

Some described the significance of predictive disease modelling to understand the COVID-19 trajectory and inform decisions, as well as to demonstrate to the public the effectiveness of particular measures, which *"help[ed] sustain our response"* (IDI2). Others, however, perceived that *"mathematical models were vastly overused [and] overvalued in decision-making around this pandemic"* (IDI1) and that modellers stepped outside their realm of expertise in providing models and policy recommendations through the public media.

Overall, while many actors’ impressions were that the response was evidence-informed, an overarching theme was a lack of clarity and uncertainty with respect to *how* evidence actually flowed into decision-making processes, as well as what specific evidence was used and how. Participants noted various mechanisms created or already in place prior to COVID-19 that fed data into, and facilitated, decision-making. There was an acknowledgement that multiple forms of evidence—including scientific data, data on public perceptions, as well as public pressure—appeared to have influenced decision-making.

### Interface between researchers and policy-makers

There was a general sense that the Ministry supported the use of scientific and research-based evidence specifically. Some actors identified particular Ministry personnel as being especially amenable to research and focused on data to inform decisions and implementation. More broadly, the government-research interface was characterized by one actor as an amicable one, a *"research-friendly government",* and that the Ministry of Health (MOH), specifically, has a research strategy whereby, *"it’s literally within their bureaucracy to become a more evidence-informed organization"* (IDI11). The MOH was noted to have funded a research network intended to channel evidence into health policy and practice, and which reported to the research side of the MOH.

Other actors perceived relatively limited engagement with the broader scientific community. Some perceived an overreliance on 'in-house expertise' or a *"we can do that [ourselves] mentality"* within government that precluded academic researchers’ involvement, as well as a sense of *"not really always wanting to engage with academics to answer policy questions because they don’t necessarily see the value that comes"* (IDI14). With respect to the role of research, an actor stated:

There needs to be a provincial dialogue around what evidence is and how it gets situated, because there’s been some tension around evidence being produced and not used or at least not used in the way that researchers think that it should be (IDI11).

Those involved in data analytics within the MOH acknowledged a challenge in making epidemiological data available to academic researchers, because *"at the time, you’re just trying to get decisions made"* (IDI7). Relatedly, a research actor described the rapid instigation of COVID-19 research and pivoting of academic research programs to respond to the pandemic, but perceived a slow uptake of these research efforts from the MOH and PHSA for decision-making and action. Nevertheless, they too acknowledged the challenge of using research evidence, specifically, in an evolving and dynamic pandemic:

I think we’ve got to be realistic about what research in a pandemic situation can realistically contribute within very short timelines. I mean, some of these decisions have to be made very quickly...they were intuitive decisions, I think some of them, rather than necessarily evidence-based decisions (IDI14).

Relatedly, perspectives diverged on the relationship between 'government' and public health expertise, and whether or not public health actors had an independent voice in articulating evidence to inform governance during the pandemic. Largely from Ministry stakeholders, and those within the PHSA, the impressions were that Ministry actors were relying on public health advice and scientific expertise. As one actor articulated, *"[the] government actually respected and acknowledged and supported public health expertise"* (IDI9). Others emphasized a *"trust of the people who understood the problem"* (IDI3)—namely, those within public health—and perceived that public health experts were enabled *"to take a lead role in the health system, over politics"* (IDI12). This perspective was not as widely held by those in the public health sector, as one public health actor expressed, *"politicians and bureaucrats waded into public health practice in a way that I don't think was appropriate"* and that, *"in the context of a pandemic, it’s actually relatively challenging to bring true expert advice because there’s too many right now. Suddenly, everybody’s a public health expert, but especially bureaucrats and politicians."* They went on to share that the independence of public health to speak and act—and for politicians to accept independent public health advice—needs to be protected and institutionalized as *"core to good governance"* (IDI1). Relatedly, an elected official linked this to the absence of a formal, independent science table to advise government and stated that, *"I think we should have one established permanently. I think we need to recognize that politicians aren't always the best at discerning scientific evidence and how that should play into decision-making"* (IDI15).

These results highlight the divergent perspectives participants had as to the interface between research and policy-making and a lack of understanding regarding process and roles.

### Challenges in applying evidence to policy decisions

Perspectives converged with respect to the existence of numerous challenges with and barriers to applying evidence to health policy and decision-making. These related to the quality and breadth of available data, both in terms of absence and abundance. For instance, as one public health actor noted in relation to health policy-making, *"you never have enough information. You always have an information shortage, so you're trying to make the best decisions you can in the absence of usually really clear information"* (IDI8). On the other hand, as evidence emerged *en masse* across jurisdictions in the pandemic, there were challenges with synthesizing evidence in a timely fashion for 'real-time' decision-making. A regional health authority actor highlighted this challenge early in the COVID-19 pandemic and perceived that there was not a provincial group bringing new synthesized information to decision-makers on a daily basis (IDI6). Other challenges related to the complexity of the political-public health interface with respect to data and scientific expertise, which *"gets debated and needs to be digested by the political process. And then decisions are made"* (IDI5). This actor further expressed that debate among experts needs to be balanced with efficient crisis response, that one has to *"cut the debate short. For the sake of expediency, you need to react."*

It was observed that, in BC’s COVID-19 response, data was gathered from multiple sources with differing data collection procedures, and sometimes with conflicting results—for instance, 'health system data' analyzed by the PHSA and 'public health data' analyzed by the BCCDC. This was observed to present challenges from a political perspective in discerning *"who’s actually getting the 'right' answers"* (IDI7). An added layer of complexity was reportedly rooted in how to communicate such evidence to the public and *"public trust in the numbers"* (IDI7), particularly as public understanding of what evidence is, how it is developed, and why it changes, can influence public perceptions of governance.

Finally, as one actor from within the research sector noted, organizationally and governance-wise, the system was *"not very well set up to actually use research evidence…if we need to do better at using evidence in practice, we need to fix some of those things. And we actually know what a lot of those things are."* For example*, "there’s no science framework for how organizations work within that" *and "*governments shy away from setting science policy*" (IDI11). This challenge was framed as having a macro-level dimension, as higher-level leadership structures were observed to not incentivize the development and effective use of research among constituent organizations, and also micro-level implications. From their perspective, researchers will struggle without such policy frameworks to obtain necessary data-sharing agreements with health authorities, nor will they be able to successfully navigate other barriers to conducting action-oriented research that informs policy and practice.

Similarly, a research actor perceived that the COVID-19 pandemic highlighted pre-existing fragmentation, *"a pretty disjointed sort of enterprise"* in how research is organized in the province:

I think pandemics need strong leadership and I think pandemic research response needed probably stronger leadership than it had. And I think that’s to do with [how] no one really knew who was in charge because no one really was given the role of being truly in charge of the research response (IDI14).

This individual underscored that, at the time of the interview, there were nearly 600 separate research projects being conducted in BC that focused on COVID-19. From their perspective, this reflected the need for more centralized direction to provide leadership, coordinate research efforts, and catalyze collaborations.

Overall, respondents perceived a lack of coordination and continuity across data sources, and a lack of explicit guidelines on evidence-use in the decision-making process, which resulted in a sense of fragmentation. The tension between the processes involved in research and the need for rapid decision-making was perceived as a barrier to using evidence to inform policy.

## Discussion

This study explored the use of evidence to inform early COVID-19 decision-making within British Columbia, Canada, from the perspectives of decision-makers themselves. Findings underscore the complexity of synthesizing and applying evidence (i.e., ‘scientific’ or research-based evidence most commonly discussed) to support public health policy in 'real-time', particularly in the context of public health crisis response. Despite a substantial and long-established literature on evidence-based clinical decision-making [23, 24], understanding is more limited as to how public health crisis decision-making can be evidence-informed or evidence-based. By contributing to a growing global scholarship of retrospective examinations of COVID-19 decision-making processes [25–28], our study aimed to broaden this understanding and, thus, support the strengthening of public health emergency preparedness in Canada, and globally. 

Specifically, based on our findings on evidence-based public health practice, we found that decision-makers clearly emphasized ‘evidence-based’ or ‘evidence-informed’ as meaning ‘scientific’ evidence. They acknowledged other forms of evidence such as professional expertise and contextual information as influencing factors. We identified four key points related to the process of evidence-use in BC's COVID-19 decision-making, with broader implications as well:

1. **Role Differences:** The tensions we observed primarily related to a lack of clarity among the various agencies involved as to their respective roles and responsibilities in a public health emergency, a finding that aligns with research on evidence-use in prior pandemics in Canada [29]. Relatedly, scientists and policy-makers experienced challenges with communication and information-flow between one another and the public, which may reflect their different values and standards, framing of issues and goals, and language [30].
2. **Barriers to Evidence-Use:** Coordination and consistency in how data are collected across jurisdictions reportedly impeded efficiency and timeliness of decision-making. Lancaster and Rhodes (2020) suggest that evidence itself should be treated as a process, rather than a commodity, in evidence-based practice [31]. Thus, shifting the dialogue from 'barriers to evidence use' to an approach that fosters dialogue across different forms of evidence and different actors in the process may be beneficial.
3. **Use of Evidence in Public Health versus Medicine:** Evidence-based public health can be conflated with the concept of evidence-based medicine, though these are distinct in the type of information that needs to be considered. While ‘research evidence’ was the primary type of evidence used, other important types of evidence informed policy decisions in the COVID-19 public health emergency—for example, previous experience, public values, and preferences. This concurs with Brownson’s (2009) framework of factors driving decision-making in evidence-based public health [32]. Namely, that a balance between multiple factors, situated in particular environmental and organizational context, shapes decision-making: 1) best available research evidence; 2) clients'/population characteristics, state, needs, values, and preferences; and 3) resources, including a practitioner’s expertise. Thus, any evaluation of evidence-use in public health policy must take into consideration this multiplicity of factors at play, and draw on frameworks specific to public health [33]. Moreover, public health decision-making requires much more attention to behavioural factors and non-clinical impacts, which is distinct from the largely biology-focused lens of evidence-based medicine.
4. **Transparency:** Many participants emphasized a lack of explanation about why certain decisions were made and a lack of understanding about who was involved in decisions and how those decisions were made. This point was confirmed by a recent report on lessons learned in BC during the COVID-19 pandemic in which the authors describe "*the desire to know more about the reasons why decisions were taken*" as a "*recurring theme*" (13:66). These findings point to a need for clear and transparent mechanisms for channeling evidence, irrespective of the form used, into public health crisis decision-making.

Our findings also pointed to challenges associated with the infrastructure for utilizing research evidence in BC policy-making, specifically a need for more centralized authority on the research side of the public health emergency response to avoid duplication of efforts and more effectively synthesize findings for efficient use. Yet, as a participant questioned, what is the realistic role of research in a public health crisis response? Generally, most evidence used to inform crisis response measures is local epidemiological data or modelling data [7]. As corroborated by our findings, challenges exist in coordinating data collection and synthesis of these local data across jurisdictions to inform 'real-time' decision-making, let alone to feed into primary research studies [34].

On the other hand, as was the case in the COVID-19 pandemic, a 'high noise' research environment soon became another challenge as data became available to researchers. Various mechanisms have been established to try and address these challenges amid the COVID-19 pandemic, both to synthesize scientific evidence globally and to create channels for research evidence to support timely decision-making. For instance: 1) **research networks and collaborations** are working to coordinate research efforts (e.g., COVID-END network [35]); 2) **independent research panels or committees within jurisdictions** provide scientific advice to inform decision-making; and 3) **research foundations, funding agencies, and platforms for knowledge mobilization (e.g., academic journals)** continue to streamline funding through targeted calls for COVID-19 research grant proposals, or for publication of COVID-19 research articles. While our findings describe the varied forms of evidence used in COVID-19 policy-making—beyond scientific evidence—they also point to the opportunity for further investments in infrastructure that coordinates, streamlines, and strengthens collaborations between health researchers and decision-makers that results in timely uptake of results into policy decisions.

Finally, in considering these findings, it is important to note the study's scope and limitations: We focused on evidence use in a single public health emergency, in a single province. Future research could expand this inquiry to a multi-site analysis of evidence-use in pandemic policy-making, with an eye to synthesizing lessons learned and best practices. Additionally, our sample of participants included only one elected official, so perspectives were limited from this type of role. The majority of participants were health officials who primarily referred to and discussed evidence as ‘scientific’ or research-based evidence. Further work could explore the facilitators and barriers to evidence-use from the perspectives of elected officials and Ministry personnel, particularly with respect to the forms of evidence—considered broadly—and other varied inputs, that shape decision-making in the public sphere. This could include a more in-depth examination of policy implementation and how the potential societal consequences of implementation factor into public health decision-making.

## Conclusions

We found that the policy decisions made during the initial stages of the COVID-19 pandemic were perceived by actors in BC's response as informed by—not always based on—scientific evidence, specifically; however, decision-makers also considered other contextual factors and drew on prior pandemic-related experience to inform decision-making, as is common in evidence-based public health practice [32]. The respondents' experiences point to specific areas that need to be considered in planning for future public health emergencies, including information flow between policy-makers and researchers, coordination in how data are collected, and transparency in how decisions are made—all of which reflect a need to improve communication. Furthermore, shifting the discourse from evidence as a commodity to evidence-use as a process will be helpful in addressing barriers to evidence-use, as well as increasing understanding about the public health decision-making process as distinct from clinical medicine. Finally, there is a critical need for clear mechanisms that channel evidence (whether ‘scientific’, research-based, or otherwise) into health crisis decision-making, including identifying and communicating the decision-making process to those producing and synthesizing evidence. The COVID-19 pandemic experience is an opportunity to reflect on what needs to be done to guild our public health systems for the future [36, 37]. Understanding and responding to the complexities of decision-making as we move forward, particularly with respect to the synthesis and use of evidence, can contribute to strengthening preparedness for future public health emergencies.

### Supplementary Information

**Additional file 1. **Semi-structured interview guide [* = questions used for this specific study]

## Data Availability

The data that support the findings of this study are not publicly available to maintain the confidentiality of research participants.

## Abbreviations

BC: British Columbia
BCCDC: British Columbia Centre for Disease Control
COVID-19: Coronavirus Disease 2019
MHO: Medical Health Officer
MOH: Ministry of Health
PHO: Provincial Health Officer
PHSA: Provincial Health Services Authority
SARS-CoV-2: Severe Acute Respiratory Syndrome Coronavirus—2
UBC: University of British Columbia

## Footnotes

1 The terms *'evidence-informed'* and *'evidence-based'* decision-making are used throughout this paper, though are distinct. The term *'evidence-informed'* suggests that evidence is used and considered, though not necessarily solely determinative in decision-making [38].

2 The Provincial Health Services Authority (PHSA) works with the Ministry of Health (MOH) and regional health authorities to oversee the coordination and delivery of programs.

3 The Office of the Provincial Health Officer (PHO) has binding legal authority in the case of an emergency, and responsibility to monitor the health of BC’s population and provide independent advice to Ministers and public offices on public health issues.

4 The British Columbia Centre for Disease Control (BCCDC) is a program of the PHSA and provides provincial and national disease surveillance, detection, treatment, prevention, and consultation.
