# Supplementary material for: The use of evidence to guide decision-making during the COVID-19 pandemic: divergent perspectives from a qualitative case study in British Columbia, Canada
Source: Health Res Policy Syst. 2024 Jun 3;22:66. doi: 10.1186/s12961-024-01146-2 (PMC11145826; doi:10.1186/s12961-024-01146-2)
Supplement: Supplementary file 1 — Additional file 1. Semi-structured interview guide [* = questions used for this specific study] [file 12961_2024_1146_MOESM1_ESM.docx]

**Additional files**

**Additional file 1 –** Semi-structured interview guide *[* = questions used for this specific study]*

**Background/context questions:**

1. Please describe your professional background and titles and positions (in which organizations) relevant to the COVID-19 pandemic response in the period leading up to March 18^th^ 2020.
2. How did you come to serve in those positions? (For example, how were you appointed and recruited and by whom?). To whom did you report in those positions?

**Questions on activities leading up to March 18^th^ declaration of a state of emergency:**

1. At what point did you realize the seriousness of the new virus? What evidence led you to realize it was serious?*
2. When did you first hear about discussions in BC considering declaring a state of emergency? Please describe how you were informed about that and how it related to your role and position at the time.
3. Were you personally involved in discussions about when and how to declare a state of emergency in BC? In what ways were you involved?
4. What information was used to make the decision? What was the reasoning behind the decision?*
5. Who else was involved in the decision-making procedure? What were their roles and positions?
6. Were there other processes that you heard about or were informed about but not participate in personally? Please describe those as well.
7. What were your perceptions about the role or impact of federal-level decisions on these processes?

**Questions on activities following the March 18^th^ declaration of a state of emergency:**

1. How did your role and work change as a result of the declaration? For example, did the organization employing you change? Did your supervision or reporting relationships change? Did your direct supervisory role change? Were you given any new tasks and resources?
2. Several specific orders were launched under the authority of the declaration. For example, schools were closed as were businesses with liquor licenses. In the next few days other businesses were closed and border closings occurred. What was your role in relation to these orders? With whom did you work or collaborate? What influenced those decisions?

**Questions on roles and participation in other key decision points after March 2020**

***Context:*** *In the months following March 2020, BC advanced from Phase 1 restrictions to Phase 2 (May 2020) and Phase 3 (July 2020). In November 2020, BC introduced some additional restriction without declaring another phase. We identified one or two decision points relevant to the participant’s role in the period between May 2020 and today.*

1. How did your role and position change during this time?
2. What was your role specifically in relation to the decision point? Please describe in some detail regarding people you worked with and your observations about the decision processes. How were these different from the earlier period? What information informed these decisions?*
3. Were you involved in some way with efforts in January to develop a test for the virus? If so, please describe how you were involved.

**Questions on overall perception of process:**

1. Considering these different decision points in the evolution of the COVID-19 pandemic in BC, how would you describe your main engagement or contribution? Please mention different engagements and describe them. For example:*
2. Analyst and provider of scientific evidence to decision makers;
3. Participant in decision-making committees or high-level consultations
4. Advisor to senior decision-maker
5. Give us your views on how well scientific evidence was heard and attended to?*
6. In your engagement or contributions during that time, were you directly meeting with or communicating with persons holding political office, such as elected officials? Please describe some examples.
